# Differences in pharmacologic and demographic factors in male and female patients with vascular dementia, Alzheimer's disease, and mixed vascular dementia

**DOI:** 10.3389/frdem.2023.1137856

**Published:** 2023-06-30

**Authors:** Madison Stanley, Nicolas Poupore, Krista Knisely, Alyssa Miller, Adebobola Imeh-Nathaniel, Laurie Theriot Roley, Samuel Imeh-Nathaniel, Rich Goodwin, Thomas I. Nathaniel

**Affiliations:** ^1^School of Medicine Greenville, University of South Carolina, Greenville, SC, United States; ^2^Department of Biology, North Greenville University, Tigerville, SC, United States; ^3^Prisma Health, Greer, SC, United States

**Keywords:** Alzheimer's disease, vascular dementia (VaD), mixed vascular dementia, dementia, male and female

## Abstract

**Background:**

Increasing evidence suggests that demographic and pharmacologic factors may play a significant role in the epidemiology of dementia. Sex differences in prevalence also depend on dementia subtypes, such as Alzheimer's dementia (AD), vascular dementia (VaD), and mixed vascular-Alzheimer's dementia (MVAD). Therefore, studies are needed to investigate sex-specific differences, and identify potential therapeutic targets for both sexes.

**Methods:**

Data was collected from the Prisma Health-Upstate Alzheimer's registry from 2016 to 2021 for 6,039 VaD patients, 9,290 AD patients, and 412 MVAD patients. A logistic regression was used to determine demographic and pharmacological factors associated with gender differences in patients with VaD, AD, and MVAD.

**Results:**

In patients with VaD, African Americans (OR = 1.454, 95% CI, 1.257–1.682, *p* < 0.001) with increasing age (OR = 1.023, 95% CI, 1.017–1.029, *p* < 0.001), treated with aripiprazole (OR = 4.395, 95% CI, 2.880–6.707, *p* < 0.001), were associated with females, whereas patients treated with galantamine (OR = 0.228, 95% CI, 0.116–0.449, *p* < 0.001), memantine (OR = 0.662, 95% CI, 0.590–0.744, *p* < 0.001), with a history of tobacco (OR = 0.312, 95% CI, 0.278–0.349, *p* < 0.001), and ETOH (OR = 0.520, 95% CI, 0.452–0.598, *p* < 0.001) were associated with males. Among AD patients, African Americans (OR = 1.747, 95% CI, 1.486–2.053, *p* < 0.001), and Hispanics (OR = 3.668, 95% CI, 1.198–11.231, *P* = 0.023) treated with buspirone (OR = 1.541, 95% CI, 1.265–1.878, *p* < 0.001), and citalopram (OR = 1.790, 95% CI, 1.527–2.099, *p* < 0.001), were associated with females, whereas patients treated with memantine (OR = 0.882, 95% CI, 0.799–0.974, *p* = 0.013), and with a history of tobacco (OR = 0.247, 95% CI, 0.224–0.273, *p* < 0.001), and ETOH (OR = 0.627, 95% CI, 0.547–0.718, *p* < 0.001) were associated with male AD patients. In patients with MVAD, rivastigmine (OR = 3.293, 95% CI, 1.131–9.585, *p* = 0.029), memantine (OR = 2.816, 95% CI, 1.534–5.168, *p* < 0.001), and risperidone (OR = 10.515, 95% CI, 3.409–32.437, *p* < 0.001), were associated with females while patients with an increased length of stay (OR = 0.910, 95% CI, 0.828–1.000, *p* = 0.049), with a history of tobacco (OR = 0.148, 95% CI, 0.086–0.254, *p* < 0.001) and ETOH use (OR = 0.229, 95% CI, 0.110–0.477, *p* < 0.001) were more likely to be associated with males.

**Conclusions:**

Our study revealed gender differences and similarities in the demographic and pharmacological factors of VaD, AD, and MVAD. Prospective studies are needed to determine the role of demographic and pharmacological factors in reducing sex-based disparities among VaD, AD, and MVAD patients.

## 1. Introduction

Vascular dementia (VaD), Alzheimer's disease (AD), and Mixed Vascular-Alzheimer's Dementia (MVAD) are neurodegenerative cognitive disorders that have a variety of effects on each individual (Attems and Jellinger, 2014). Vascular dementia is commonly caused by a reduced blood flow to the cerebrum (Song et al., 2014), which may lead to neuronal death, brain atrophy, and neurocognitive decline (Ashraf et al., 2014). The main contributors to VaD include stroke, cardiovascular disease (CVD), hyperlipidemia, and hypertension (Song et al., 2014). The pathology of AD results from the accumulation of misfolded β-amyloid proteins and neurofibrillary tangles of hyperphosphorylated tau proteins (Ashraf et al., 2014). While dementia in the elderly population is a continuum of pathologies (Serrano-Pozo et al., 2013), AD and VaD represent the two extremes, and “mixed” dementia lies in the middle and comprises most cases (Lambert et al., 2018). The coexistence of AD and cerebrovascular disease in a patient is diagnosed as MVAD (Kalaria, 2002). Both MVAD and AD share components of cerebral vascular degeneration and accumulation of proteins (Podcasy and Epperson, 2016). This poses a challenge in the diagnosis and treatment of MVAD to clinicians, and most diagnostic procedures are biased toward AD (Custodio et al., 2017).

AD disproportionately affects more females than males (Coker-Ayo et al., 2022), and this is frequently attributed to the longer average lifespan for females (Carter et al., 2002) while there has been no clear connection to sex differences in VaD patients. While some studies (Andersen et al., 1999) suggest no sex difference, others (Podcasy and Epperson, 2016) reported a higher incidence of VaD in males than females. Patients with MVAD can either present with AD symptoms and have cerebrovascular lesions detected at autopsy or have combined clinical features of both AD and VaD (Magaki et al., 2014). The only definitive way to diagnose MVAD is by performing an autopsy, making it challenging to study gender differences in living patients. Retrospective data allows for the identification of demographic factors that contribute to sex differences in patients with AD, VaD, or MVAD.

Pharmacologic treatment of dementia is complex (Karakaya et al., 2013), with a majority of treatments focused on the management of AD and related symptoms. The Diagnostic and Statistical Manual for Mental Disorders (DSMMD) divides cognitive function into five domains: (1) learning and memory, (2) language, (3) visuospatial, (4) executive, and (5) psychomotor. An impairment in at least two domains is necessary to diagnose dementia (Tarawneh and Holtzman, 2012). Pharmacologic strategies target the impaired domain attenuating symptoms by reducing the breakdown of acetylcholine and increasing the concentration of neurotransmitters in AD patients (Solomon et al., 2011; Perera et al., 2014). Central acetylcholinesterase inhibitors (AChEIs), donepezil, galantamine, and rivastigmine, are considered to manage the cognitive decline in AD patients (Mehta et al., 2012). In addition, second-generation antipsychotics (SGAs), such as aripiprazole, olanzapine, risperidone, and selective serotonin reuptake inhibitors (SSRIs), including citalopram, escitalopram, and paroxetine, have been used to manage behavioral and psychological conditions (Wang et al., 2016; Kim et al., 2018; Ruberto et al., 2020). Additional pharmacotherapies include memantine, trazodone, buspirone, and valproate (Hersch and Falzgraf, 2007; Madhusoodanan and Ting, 2014; Coker-Ayo et al., 2022).

The different pharmacologic strategies for dementia in AD have shown limited or no effect on patients with other types of dementia, including VaD (Perera et al., 2014), and MVAD (Langa et al., 2004). The therapeutic decision to treat AD, VaD, or MVAD patients with AChEIs is based on the likelihood that AD is the underlying etiology of the patient's symptoms (Grossberg et al., 2019). In addition, other medications can be combined with an AChEI to treat additional symptoms, including the ones in AD, VaD, and MVAD patients.

Understanding similarities and differences in the pharmacological treatments for males and females with AD, VaD, and MVAD might reveal new and improved strategies to eliminate gender differences in treating dementia for males and females. Females present with higher cognitive and functional decline rates (Tarawneh and Holtzman, 2012), and constitute more than two-thirds of AD patients in the United States (Hebert et al., 2013). Our study focused on AD, VaD, and MVAD patients, and our hypothesis is that more females than males are affected, as commonly seen in the AD population (Vina, 2010). Further, we hypothesize that males and females with AD, VaD, and MVAD differ regarding treatment with AChEIs, SSRIs, and SGAs. In addition, since the cognitive progression in AD declines at a faster rate in females compared to males (Lin et al., 2015; Laws et al., 2016), we analyzed specific demographic factors contributing to gender differences in patients who received different medications including AChEIs, SSRIs, and SGAs.

## 2. Methods

### 2.1. Study population

Data for AD, VaD, and MVAD patients were collected from the Alzheimer's registry of Prisma Health-Upstate between February 2016 and August 2021. This study was approved by the Prisma Health committee for research compliance (approval number: 00052571). Data were extracted for the patient's medications, including selective serotonin receptor inhibitors (SSRI), citalopram, escitalopram, paroxetine, and central acetylcholinesterase inhibitors (ChEI), such as donepezil, galantamine, and rivastigmine. In addition, data for second-generation antipsychotics (SGA), such as aripiprazole, olanzapine, risperidone, memantine, trazodone, buspirone, and valproate, were extracted. Data for tobacco and alcohol use, race, biologicals sex, age, and ethnicity were also collected. Inclusion factors for this study were medication history, and demographics for patients with VaD, AD, and MVAD. Patients with other forms of dementia not defined as vascular or Alzheimer's were excluded. Dosages, frequencies, and therapy length were unavailable for this cohort. Length of stay was determined from the date of admission to the date of discharge to either home, another hospital, or a long-term care facility.

### 2.2. Statistical analysis

Descriptive statistics, including frequencies of categorical variables, means and standard deviations of continuous variables were determined for VaD, AD, and MVAD patients for the following variables: sex, age, history of tobacco, ETOH, race, length of stay. We also analyzed medications including, central acetylcholinesterase inhibitors (AChEIs), donepezil, galantamine, and rivastigmine; second-generation antipsychotics (SGAs), such as aripiprazole, olanzapine, risperidone, and selective serotonin reuptake inhibitors (SSRIs), including citalopram, escitalopram, and paroxetine. All continuous variables were analyzed and presented as means, and standard deviations, while categorical variables were presented as percentages. Nominal variables were analyzed with a Pearson χ2 test, while continuous variables were determined using the student's *t*-test. For this analysis, the variables for AChEIs, SGAs, and SSRIs indicate the number of patients taking at least one medication in the medication category. Since some patients were taking more than one medication in a particular class, the AChEIs, SGAs, and SSRIs variables may be equal or less than the sum of the individual medications. In addition, since our study is non- randomized, the logistic regression analysis was used to adjust for the demographic and pharmacologic factors associated with male or female patients with VaD, AD, or MVAD.

This study also examined demographic and pharmacological factors that could be independently associated with male or female patients with VaD, AD, or MVAD. These factors were examined separately for male and female patients with VaD, AD, or MVAD with adjustment for all demographic and pharmacological factors. The adjusted analysis was performed using the backward selection method based on the likelihood ratio. This approach allowed all of the initially selected factors to be added to the model and then methodically eliminated if they did not add to the overall significance of the model. For each regression model, the dependent variable was male or female. In contrast, the independent variables were the demographic, social, and pharmacologic factors stratified by type of dementia (VaD, AD, or MVAD). Variables with an odds ratio (OR) *P*-value < 0.05 at 95% confidence intervals (95% CI) were included in the regression models to identify demographic and pharmacological factors that are independently associated with male or female VaD, AD, or MVAD patients. Multicollinearity and interactions of variables in the models were checked using the Hosmer-Lemeshow test. The area under the receiver operating curve (AUROC) was determined to test the model's sensitivity, specificity, and accuracy. All statistical analyses were performed using the Statistical Package for Social Sciences v 26.0 for Windows (SPSS, Chicago, IL).

## 3. Results

A total of 15,741 patients with either VaD, AD, or MVAD were identified in this study. Of this cohort, 6,039 presented with VaD, 9,290 presented with AD while 412 presented with MVAD (Table 1). Patients with VaD were more likely to be younger, male, African American, or Hispanic. Patients with VaD presented with higher rates of using tobacco, ETOH, had a longer length of stay and were more likely to be treated with aripiprazole, escitalopram, and buspirone. AD patients were more likely to be treated with AChEI, particularly donepezil, galantamine and rivastigmine. In addition, they were more likely to be treated with risperidone and memantine when compared with VaD and MVAD patients. MVAD patients were mostly likely to be treated with a SSRI, specifically, citalopram.

**Table 1:** Demographic and pharmacological characteristics in patients with vascular dementia, Alzheimer's disease, and mixed vascular-Alzheimer's dementia.

**Characteristic**	**Vascular dementia**	**Alzheimer's disease**	**Mixed vascular and Alzheimer's disease**	***P*-value**
**Number of patients**	**6,039**	**9,290**	**412**	
**Age group: no. (%)**				
< 50	32 (0.5)	0 (0.0)	0 (0.0)	< 0.001^*a^
50–59	166 (2.6)	0 (0.0)	0 (0.0)	
60–69	724 (11.5)	136 (1.5)	22 (5.3)	
70–79	1,707 (27.1)	1,638 (17.6)	91 (22.1)	
≥80	3,680 (58.3)	7,516 (80.9)	299 (72.6)	
Mean ± SD	80.47 ± 10.38	86.28 ± 7.41	83.70 ± 7.54	< 0.001^*b^
**Gender: no (%)**				
Male	2,806 (44.5)	2,949 (31.7)	125 (30.3)	< 0.001^*a^
Female	3,503 (55.5)	6,341 (68.3)	287 (69.7)	
**Race: no (%)**				
White	4,916 (77.9)	7,808 (84.0)	337 (81.8)	< 0.001^*a^
Black	1,187 (18.8)	1,036 (11.2)	71 (17.2)	
Other	206 (3.3)	446 (4.8)	4 (1.0)	
Hispanic ethnicity: no. (%)	71 (1.1)	179 (1.9)	3 (0.7)	< 0.001^*a^
Tobacco	3,284 (52.4)	3,898 (42.9)	203 (49.3)	< 0.001^*a^
ETOH	1,195 (19.1)	1,206 (13.2)	72 (17.5)	< 0.001^*a^
Length of Stay, day	2.52 ± 9.96	1.94 ± 4.80	1.69 ± 2.67	< 0.001^*b^
**Medications**				
Central acetylcholinesterase inhibitor	2,882 (45.7)	5,885 (63.3)	206 (50.0)	< 0.001^*a^
Donepezil	2,552 (40.5)	5,113 (55.0)	188 (45.6)	< 0.001^*a^
Galantamine	45 (0.7)	139 (1.5)	2 (0.5)	< 0.001^*a^
Rivastigmine	431 (6.8)	1,124 (12.1)	33 (8.0)	< 0.001^*a^
Second generation antipsychotic	986 (15.6)	1,487 (16.0)	71 (17.2)	0.615
Aripiprazole	176 (2.8)	209 (2.2)	5 (1.2)	0.026^*a^
Olanzapine	292 (4.6)	398 (4.3)	23 (5.6)	0.315
Risperidone	599 (9.5)	999 (10.8)	43 (10.4)	0.039^*a^
Selective serotonin receptor inhibitor	2,447 (38.8)	3,066 (33.0)	171 (41.5)	< 0.001^*a^
Citalopram	732 (11.6)	1,105 (11.9)	65 (15.8)	0.040^*a^
Escitalopram	1,783 (28.3)	2,018 (21.7)	115 (27.9)	< 0.001^*a^
Paroxetine	0 (0.0)	0 (0.0)	0 (0.0)	
Memantine	2,404 (38.1)	4,168 (44.9)	159 (38.6)	< 0.001^*a^
Trazadone	0 (0.0)	0 (0.0)	0 (0.0)	
Buspirone	560 (8.9)	671 (7.2)	15 (3.6)	< 0.001^*a^
Valproate	0 (0.0)	0 (0.0)	0 (0.0)	

Results for continuous variables are presented as Mean ± SD, while discrete data are presented as percentage frequency. Pearson's chi-square is used to compare differences in demographic and pharmacologic characteristics in patients with vascular dementia, Alzheimer's disease, and mixed vascular-Alzheimer's dementia.

^a^Pearson's Chi-Squared test.

^b^Student's *T*-test.

^*^P-value < 0.05.

Demographic and pharmacologic factors associated with sex in patients stratified by VaD, AD, or MVAD is presented in Table 2. In VaD, 2,806 were male, and 3,503 were female. Female patients with VaD were more likely to be older, African Americans, but less likely to be Hispanics. Female patients with VaD presented with lower rates of tobacco and ETOH use, had shorter lengths of stay. This group also presented with lower usage of AChEIs, specifically donepezil, galantamine, and rivastigmine, as well as SSRIs, particularly escitalopram, and memantine. However, female patients with VaD were more likely to be treated with aripiprazole and buspirone.

**Table 2:** Demographic and pharmacological characteristics of vascular dementia, Alzheimer's disease, and mixed vascular-Alzheimer's dementia stratified by sex.

	**Vascular dementia**			**Alzheimer's disease**			**Mixed vascular and Alzheimer's disease**		

**Characteristic**	**Male**	**Female**		**Male**	**Female**		**Male**	**Female**	
**Number of patients**	**2,806**	**3,503**	***P*** **-value**	**2,949**	**6,341**	***P*** **-value**	**125**	**287**	***P*** **-value**
**Age group: no. (%)**									
< 50	20 (0.7)	12 (0.3)	< 0.001^*a^	0 (0.0)	0 (0.0)	0.007^*b^	0 (0.0)	0 (0.0)	< 0.001^*a^
50–59	90 (3.2)	76 (2.2)		0 (0.0)	0 (0.0)		0 (0.0)	0 (0.0)	
60–69	385 (13.7)	339 (9.7)		32 (1.1)	104 (1.6)		2 (1.6)	20 (7.0)	
70–79	857 (30.5)	850 (24.3)		562 (19.1)	1,076 (17.0)		41 (32.8)	50 (17.4)	
≥80	1,454 (51.8)	2,226 (63.5)		2,355 (79.9)	5,161 (81.4)		82 (65.6)	217 (75.6)	
Mean ± SD	78.76 ± 10.45	81.65 ± 10.12	< 0.001^*b^	85.59 ± 7.15	86.60 ± 7.51	< 0.001^*b^	84.15 ± 6.50	83.51 ± 7.95	0.426
**Race: no (%)**									
White	2,235 (79.7)	2,681 (76.5)	0.007^*a^	2,590 (87.8)	5,218 (82.3)	< 0.001^*a^	106 (84.8)	231 (80.5)	0.049^*a^
Black	494 (17.6)	693 (19.8)		246 (8.3)	790 (12.5)		16 (12.8)	55 (19.2)	
Other	77 (2.7)	129 (3.7)		113 (3.8)	333 (5.3)		3 (2.4)	1 (0.3)	
Hispanic ethnicity: no. (%)	45 (1.6)	26 (0.7)	0.001^*a^	31 (1.1)	148 (2.3)	< 0.001^*a^	3 (2.4)	0 (0.0)	0.008^*a^
Tobacco	1,913 (68.7)	1,371 (39.4)	< 0.001^*a^	1,879 (65.5)	2,019 (32.5)	< 0.001^*a^	88 (70.4)	115 (40.1)	< 0.001^*a^
ETOH	708 (25.5)	487 (14.0)	< 0.001^*a^	526 (18.2)	680 (10.9)	< 0.001^*a^	31 (24.8)	41 (14.3)	0.010^*a^
Length of stay	2.93 ± 14.06	2.20 ± 4.60	0.009^*b^	2.13 ± 6.88	1.84 ± 3.42	0.030^*b^	2.23 ± 3.20	1.46 ± 2.37	0.016^*b^
**Medications**									
Central acetylcholinesterase inhibitor	1,399 (49.9)	1,483 (42.3)	< 0.001^*a^	1,902 (64.5)	3,983 (62.8)	0.117	58 (46.4)	148 (51.6)	0.335
Donepezil	1,216 (43.3)	1,336 (38.1)	< 0.001^*a^	1,657 (56.2)	3,456 (54.5)	0.128	55 (44.0)	133 (46.3)	0.661
Galantamine	31 (1.1)	14 (0.4)	< 0.001^*a^	62 (2.1)	77 (1.2)	0.001^*b^	2 (1.6)	0 (0.0)	0.032^*a^
Rivastigmine	238 (8.5)	193 (5.5)	< 0.001^*a^	342 (11.6)	782 (12.3)	0.312	5 (4.0)	28 (9.8)	0.048^*a^
Second generation antipsychotic	436 (15.5)	550 (15.7)	0.860	470 (15.9)	1,017 (16.0)	0.902	13 (10.4)	58 (20.2)	0.015^*a^
Aripiprazole	33 (1.2)	143 (4.1)	< 0.001^*a^	34 (1.2)	175 (2.8)	< 0.001^*a^	5 (4.0)	0 (0.0)	< 0.001^*a^
Olanzapine	143 (5.1)	149 (4.3)	0.113	131 (4.4)	267 (4.2)	0.608	4 (3.2)	19 (6.6)	0.164
Risperidone	281 (10.0)	318 (9.1)	0.207	318 (10.8)	681 (10.7)	0.950	4 (3.2)	39 (13.6)	0.002^*a^
Selective serotonin receptor inhibitor	1,132 (40.3)	1,315 (37.5)	0.023^*a^	806 (27.3)	2,260 (35.6)	< 0.001^*a^	28 (22.4)	143 (49.8)	< 0.001^*a^
Citalopram	319 (11.4)	413 (11.8)	0.603	286 (9.7)	819 (12.9)	< 0.001^*a^	2 (1.6)	63 (22.0)	< 0.001^*a^
Escitalopram	834 (29.7)	949 (27.1)	0.021^*a^	529 (17.9)	1,489 (23.5)	< 0.001^*a^	26 (20.8)	89 (31.0)	0.034^*a^
Paroxetine	0 (0.0)	0 (0.0)		0 (0.0)	0 (0.0)		0 (0.0)	0 (0.0)	
Memantine	1,220 (43.5)	1,184 (33.8)	< 0.001^*a^	1,385 (47.0)	2,783 (43.9)	0.006^*a^	33 (26.4)	126 (43.9)	< 0.001^*a^
Trazadone	0 (0.0)	0 (0.0)		0 (0.0)	0 (0.0)		0 (0.0)	0 (0.0)	
Buspirone	186 (6.6)	374 (10.7)	< 0.001^*a^	170 (5.8)	501 (7.9)	< 0.001^*a^	3 (2.4)	12 (4.2)	0.375
Valproate	0 (0.0)	0 (0.0)		0 (0.0)	0 (0.0)		0 (0.0)	0 (0.0)	

Results for continuous variables are presented as Mean ± SD, while discrete data are presented as percentage frequency. Pearson's chi-square is used to compare sex differences between demographic and pharmacological characteristics in vascular dementia, Alzheimer's disease, and mixed vascular-Alzheimer's dementia.

^a^Pearson's chi-squared test.

^b^Student's *T*-test.

^*^P-value < 0.05.

As shown in Table 2, of patients with AD, 2,949 were male, and 6,341 were female. Female patients with AD were more likely to be older African Americans or Hispanics. Female patients with AD presented with higher rates in the use of tobacco and ETOH use and shorter lengths of stay than males with AD. This group was more likely to be prescribed a SSRI, specifically citalopram and escitalopram, as well as aripiprazole and buspirone. In contrast, female patients with AD were less likely to be treated with galantamine and memantine. Of the 412 patients with MVAD, 125 were male, and 287 were females. Females with MVAD were more likely to be African American or Hispanic. Compared to males, fewer MVAD females were likely to have history of tobacco and ETOH use, and had shorter lengths of stay in the hospital. Females with MVAD were more likely to be treated with risperidone, citalopram, escitalopram, memantine, and rivastigmine but less likely to be prescribed galantamine and aripiprazole when compared to males with MVAD.

**Figure 1:**
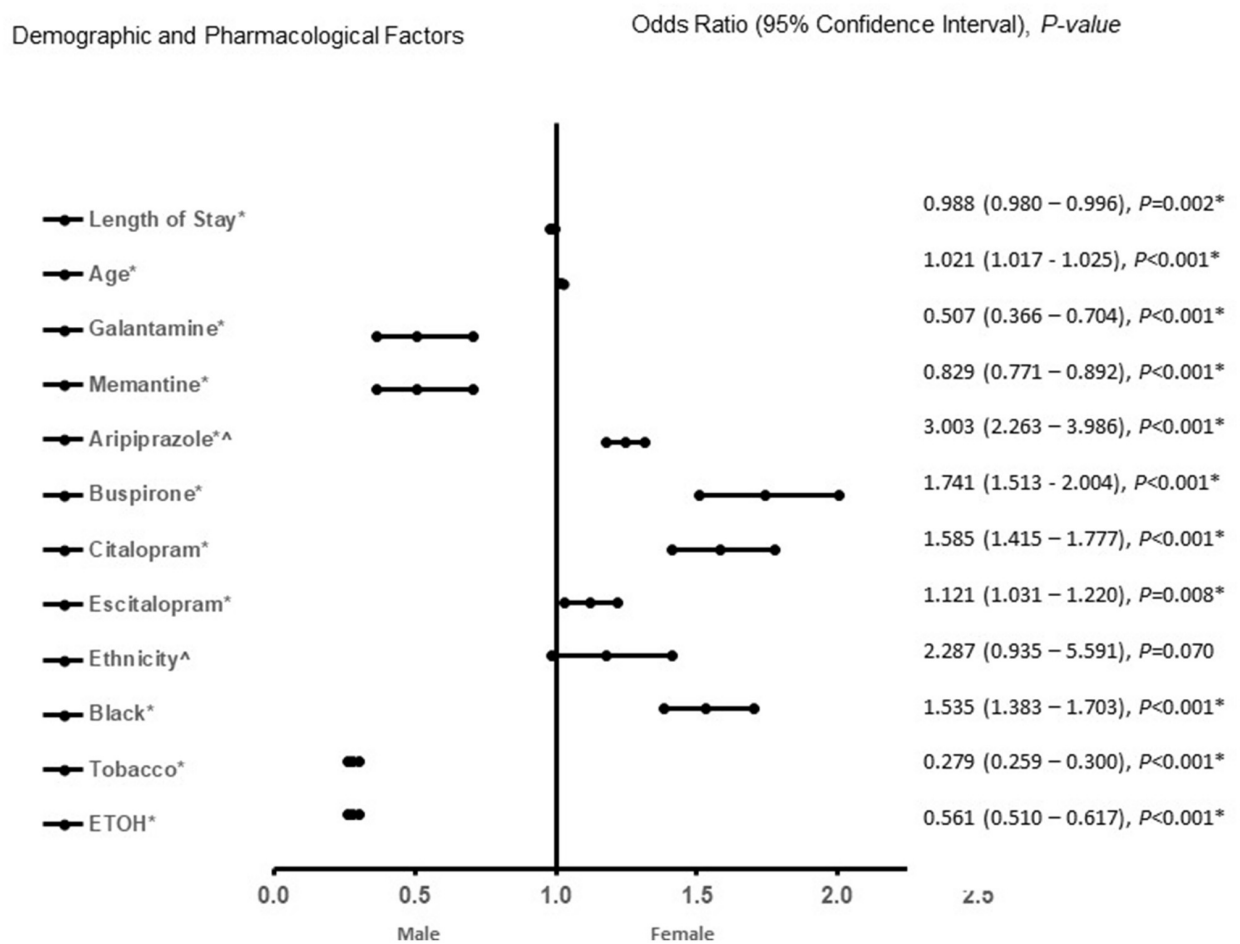
Demographic and pharmacological factors that were associated with male or female independent of type of dementia (vascular dementia, Alzheimer's disease, and mixed vascular-alzheimer's dementia). Adjusted OR < 1 denote factors that are associated with male while OR > 1 denote factors that are associated with female patients. Hosmer-Lemeshow test (*P* < 0.001*), Cox & Snell *(R*^2^ = 0.125). The overall classified percentage of 68.3% was applied to check for fitness of the logistic regression model. *Indicates statistical significance (*P* < 0.05) with a 95% confidence interval. ^∧^Indicates data were visually adjusted to fit on graph by taking 5th square root.

In the adjusted analysis, African Americans with increasing age, treated with aripiprazole, citalopram, and escitalopram were associated with females in all patients, independent of dementia pathology (Figure 1). Males were more likely to present with increased length of stay, to be treated with galantamine, memantine, and have a history of tobacco, and ETOH use. The discriminating capability of the model was moderately strong, as shown by the ROC curve, with the area under the curve (AUROC) = 0.713 (95% CI, 0.705–0.722, *P* < 0.001).

**Figure 2:**
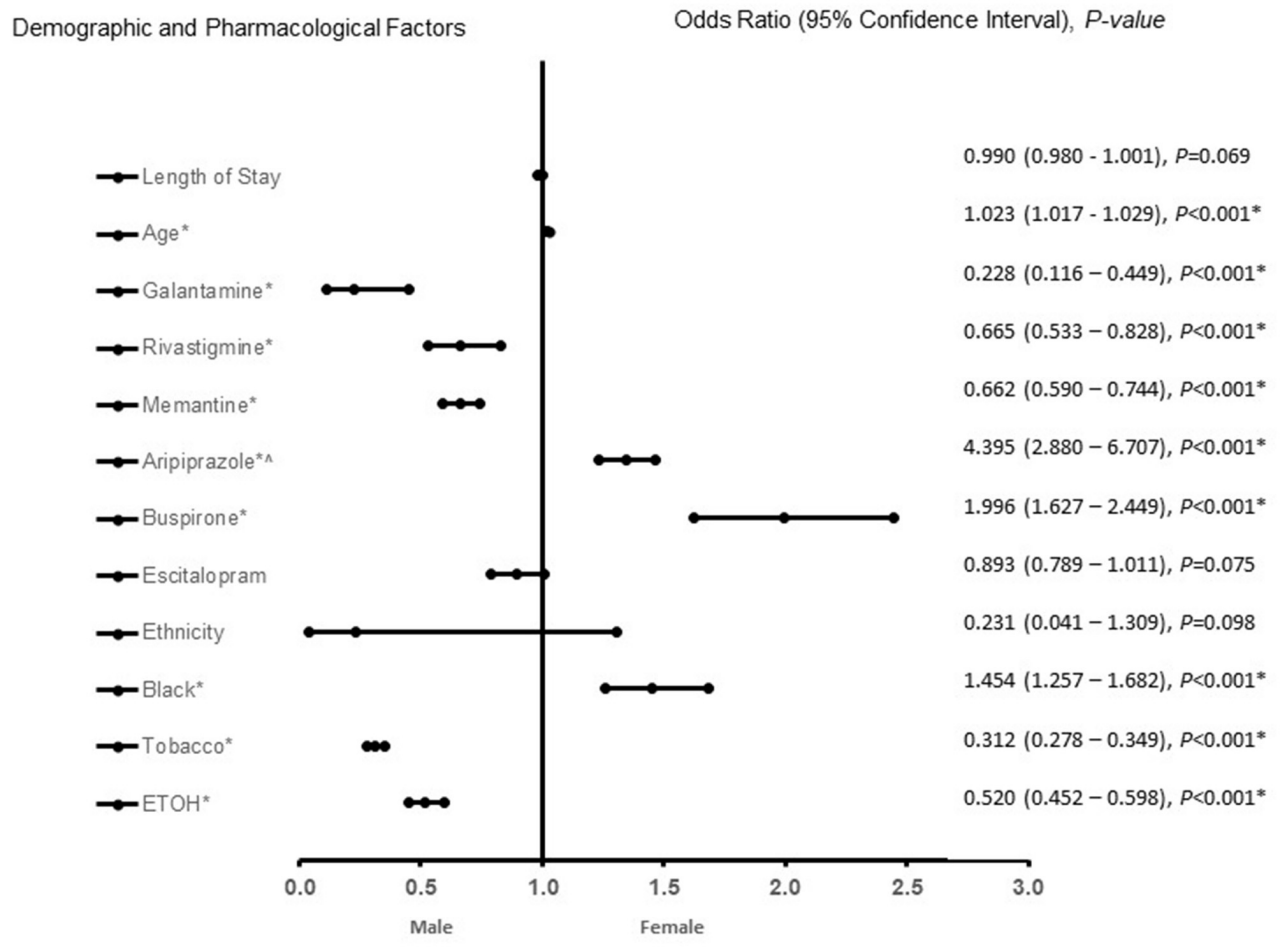
Demographic and pharmacological factors that were associated with being female in vascular dementia patients. Adjusted OR < 1 denote factors that are associated with male while OR > 1 denote factors that are associated with female patients. Hosmer-Lemeshow test (*P* < 0.001*), Cox & Snell *(R*^2^ = 0.269). The overall classified percentage of 66.8% was applied to check for fitness of the logistic regression model. *Indicates statistical significance (*P* < 0.05) with a 95% confidence interval. ^∧^Indicates data were visually adjusted to fit on graph by taking 5th square root.

In patients with VaD (Figure 2), African Americans with increasing age, treated with aripiprazole, and buspirone were more likely to be associated with females. In contrast, treatment with galantamine, rivastigmine, memantine, ETOH and tobacco use were associated with male patients with VaD. The predictive power of the logistic regression was moderately strong. The area under the curve (AUROC) is 0.718 (95% CI, 0.706–0.731, *P* < 0.001).

**Figure 3:**
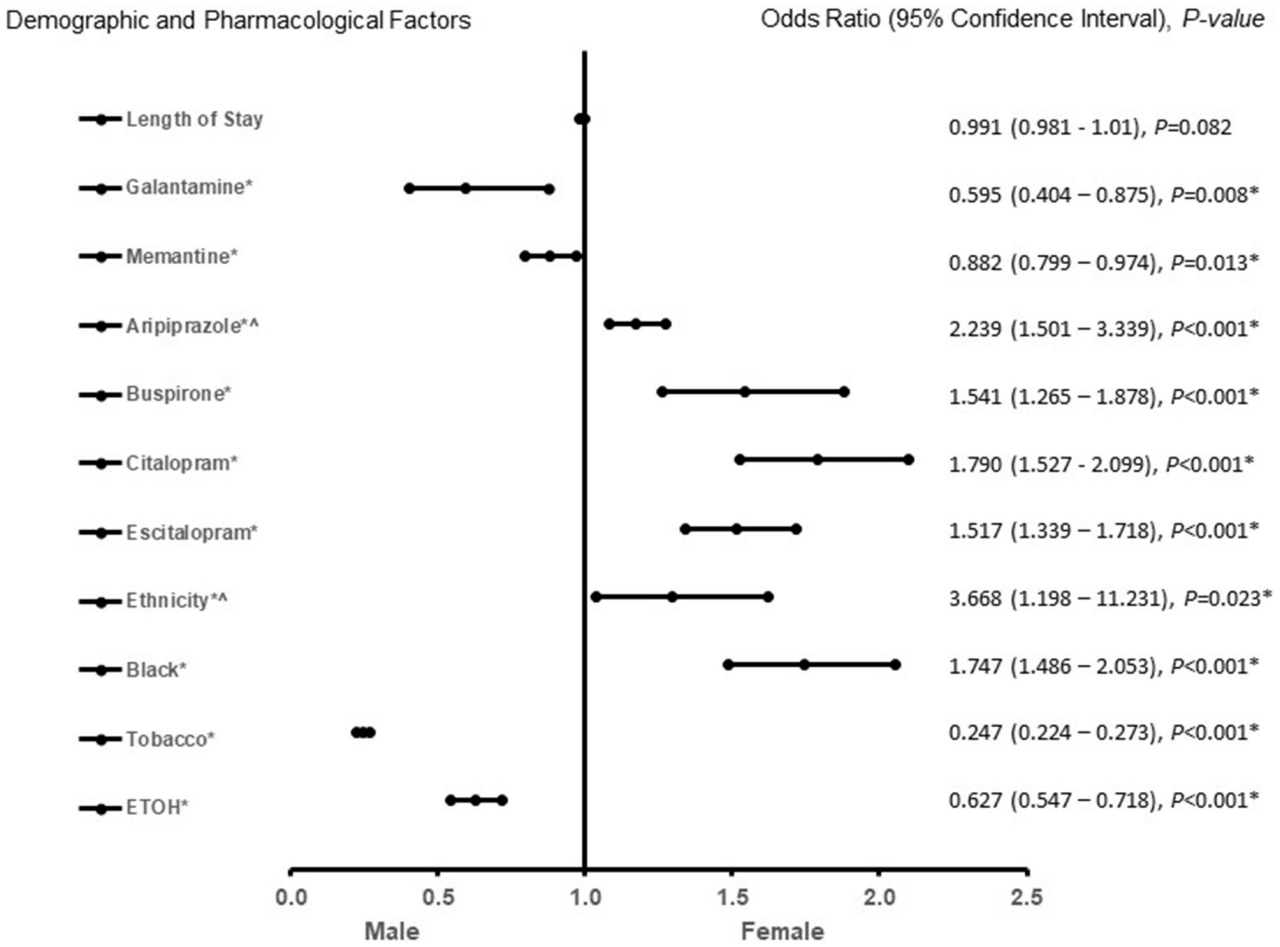
Demographic and pharmacological factors that were associated with Alzheimer's disease. Adjusted OR < 1 denote factors that are associated with male while OR > 1 denote factors that are associated with female. Hosmer-Lemeshow test (*P* < 0.001*), Cox & Snell *(R*^2^ = 0.119). The overall classified percentage of 72.5% was applied to check for fitness of the logistic regression model. *Indicates statistical significance (*P* < 0.05) with a 95% confidence interval. ^∧^Indicates data were visually adjusted to fit on graph by taking 5th square root.

**Figure 4:**
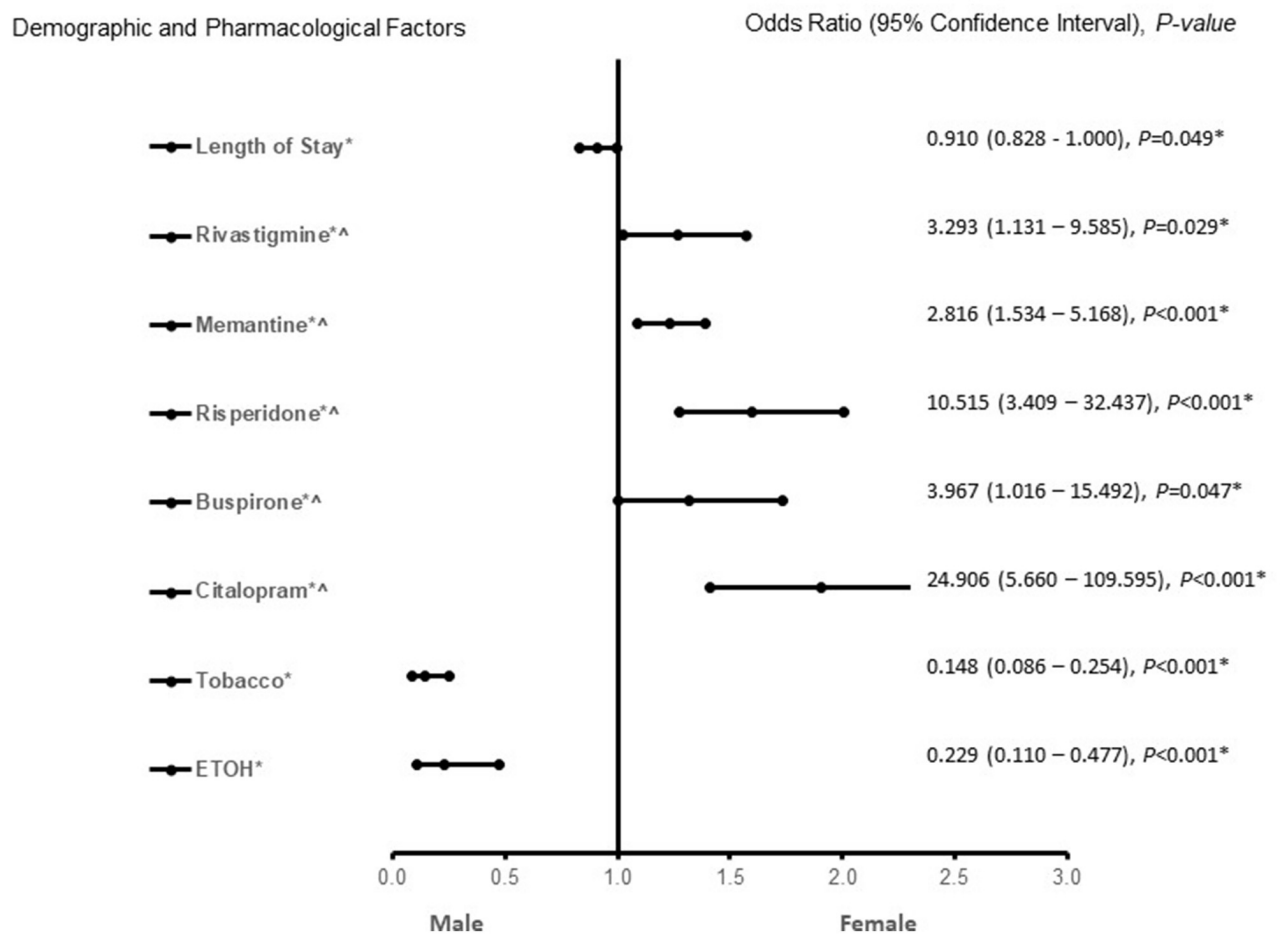
Demographic and pharmacological factors that were associated with being with mixed vascular-Alzheimer's dementia. Adjusted OR < 1 denote factors that are associated with male while OR > 1 denote factors that are associated with female patients. Hosmer-Lemeshow test (*P* = 0.016*), Cox & Snell *(R*^2^ = 0.286). The overall classified percentage of 80.8% was applied to check for fitness of the logistic regression model. *Indicates statistical significance (*P* < 0.05) with a 95% confidence interval. ^∧^Indicates data were visually adjusted to fit on graph by taking 5th square root.

In patients with AD (Figure 3), African American or Hispanic females were more likely to be treated with aripiprazole, buspirone, citalopram, and escitalopram. Galantamine, memantine, tobacco, and ETOH use were associated with males in patients with AD. The predictive power of the logistic regression was moderately strong. The area under the curve (AUROC) is 0.710 (95% CI, 0.698–0.722, *P* < 0.001). In patients with MVAD (Figure 4), females were found to be associated rivastigmine, memantine, risperidone, buspirone, and citalopram treatment. Males with MVAD were more associated with an increased length of stay and tobacco and ETOH use. The predictive power of the logistic regression was strong. The area under the curve (AUROC) is 0.842 (95% CI, 0.800–0.884, *P* < 0.001).

## 4. Discussion

With an increase in AD, VaD, and MVAD cases, researchers have taken an interest in the risk factors associated with their epidemiology. Unfortunately, an overlooked factor is often sex differences. In this study, we investigated the effect of demographic and pharmacologic factors on the reported biological sex among VaD, AD, and MVAD patients. Six major findings originated from this study. First, female patients with VaD are associated with increasing age, use of aripiprazole, buspirone, and race. Specifically, African American patients showed a stronger correlation with females. In contrast, treatment with galantamine, rivastigmine, memantine, tobacco, and ETOH use were associated with male patients with VaD. Second, treatment with aripiprazole, buspirone, and being African American, which were associated with female VaD patients, were sustained in female AD patients. Third, Female AD patients were more likely to have a Hispanic background and be prescribed citalopram or escitalopram. In both male VaD patients, galantamine, memantine, tobacco, and ETOH use displayed a strong association. Fourth, buspirone was associated with female AD patients, and the effect was sustained in female MVAD patients. Treatments with rivastigmine and memantine were associated with male VaD patients, and the effect was sustained in female MVAD patients. Firth, the effect of citalopram was significant for female VaD patients and was also found in female MVAD patients. In contrast, risperidone, which was not significant for female AD or VaD patients, was associated with female MVAD patients. Finally, both ETOH and tobacco use were significant factors in male patients with AD, VaD, and MVAD. Additionally, the length of hospital stay, which was not significant for male or female AD and VaD patients, was significant in male MVAD patients. Findings from this study indicate similarities and differences in the pharmacological treatments and demographic factors for males and females with AD, VaD, and MVAD.

There has been recent recognition that dementia in the elderly is a continuum of pathologies, with pure AD and VaD representing the two extremes and “mixed” dementia in between and possibly comprising the majority of cases (DeTure and Dickson, 2019). “Mixed” dementia is rarely diagnosed in the clinic, however, most diagnostic procedures are biased toward diagnosing AD (DeTure and Dickson, 2019). Therefore, identifying the overlap as well as some of the differences in both demographic and pharmacologic treatments in male and female patients is significant.

We observed that males with AD and VaD were treated with galantamine, while females with MVAD only received rivastigmine indicating differences in the use of ChEIs to treat males and females with AD, VaD, and MVAD. Pharmacologic treatments for AD with galantamine, and rivastigmine include targeting the primary symptoms of cognitive impairments and this is observed in both VaD and MVAD patients (Birks and Harvey, 2018; Bailey-Taylor et al., 2022). In general, ChEIs decrease acetylcholine breakdown in the brain and are used in the treatment of AD, ChEIs also offer a feasible therapeutic target to stabilize cognitive functions (Stanciu et al., 2019). Rivastigmine is a brain-selective inhibitor of acetylcholinesterase (AChE), and its metabolism is independent of cytochrome P450 (Li et al., 2019). Galantamine is a cholinergic drug that counteracts AD by reversibly inhibiting AChE and reducing central cholinergic neurotransmission (Li et al., 2019).

While galantamine and rivastigmine are reported to slow the decline in cognition and improve cognition (Li et al., 2019; Agbomi et al., 2022), rivastigmine may be slightly more efficacious than galantamine (Siddique et al., 2022) among female AD, VaD, and MVAD patients. The pharmacokinetics and pharmacodynamics of drugs vary in females and males (Soldin and Mattison, 2009). Sex-specific factors including diet, concurrent medications, and hormonal transitions are major determinants in pharmacokinetics and the pharmacodynamics of drugs in male and female patients (Beierle et al., 1999; Zucker and Prendergast, 2020). Moreover, neurotransmitter levels reduce with age at different rates in older females than in males (Barth et al., 2015). Our finding of differences in the use of ChEIs as a treatment option for males and females with AD, VaD, and MVAD suggests the need for the development of sex-specific treatment regimens to optimize outcomes in using ChEIs for male and female AD, VAD, and MVAD patients.

Risperidone was not found to be significant for female AD or VaD patients, but significant in female MVAD patients. Female patients with AD, VaD, and MVAD showed a correlation with aripiprazole and buspirone. Medications such as, aripiprazole and risperidone, are widely used as a first-line pharmacological approach to treat dementia-related psychiatric symptoms. Buspirone is a serotonin-norepinephrine reuptake inhibitor (SNRIs) and is commonly used because of its tolerability and safety profile (Crocco et al., 2017; Coker-Ayo et al., 2022). Our finding that females with AD, VaD, and MVAD were more likely to be treated with aripiprazole, buspirone or risperidone reveals a robust approach of using an SGA and SSR in treating female AD, VaD, and MVAD patients.

A major finding in this study is that female VaD and MVAD patients were treated with citalopram and escitalopram. Escitalopram is a specific selective serotonin reuptake inhibitor (SSRI) and it is a therapeutically active S-enantiomer of citalopram (Waugh and Goa, 2003). Both medications are SSRIs that inhibit the serotonin transporters (SERT), to maintain low levels of the peptide and reduce or eliminate toxic Aβ species from the brain. Accumulation of amyloid-β (Aβ) as toxic oligomers and amyloid plaques within the brain represents the pathogenic events in AD (Murphy and LeVine, 2010). One therapeutic approach is to reduce Aβ levels to limit its accumulation (Schenk et al., 2012). The administration of selective serotonin reuptake inhibitors (SSRIs) reduces brain Aβ levels in transgenic mice (Cirrito et al., 2020). Acute treatment with citalopram reduced Aβ levels by more than 20%, while chronic administration of citalopram reduced brain Aβ_40_ and Aβ_42_ levels by more than 25% (Cirrito et al., 2011). Therefore, serotonin signaling acutely reduced brain Aβ levels and chronically reduced Aβ plaques in a mouse model of AD. Therefore, serotonin-mediated activation of extracellular regulated kinase (ERK) was necessary for the regulation (Cirrito et al., 2011). The dose of each citalopram administered in mice was within the range used to treat human patients (Reagan-Shaw et al., 2008). Human PET imaging of amyloid plaques indicated that serotonin signaling was associated with less Aβ accumulation in cognitively normal individuals (Cirrito et al., 2011). Animal studies found that males and females differ in their response to citalopram exposure (Kellner and Olsén, 2020). Women are reported to have a better response to the SSRI citalopram than men, which may be due to sex-specific biological differences particularly in serotonergic systems (Young et al., 2009). The sex differences in response to citalopram, acts predominantly on serotonergic systems, and may be related to differences in the biology of men and women, particularly with respect to the role of estrogen on serotonergic systems (Young et al., 2009; Gougoulaki et al., 2021). While we cannot use the results of the current study to address mechanisms by which men and women differ in responsiveness to citalopram, we can only speculate possible differences that may account for this differential response. For example, the differences was observed despite a higher baseline severity of depression in women and no sex differences in side effect or intensity was observed. Sex differences in response to citalopram was not attributed to differences in dose of citalopram. Moreover, differences in treatment regimen were not clinically and significantly different as well. Therefore, the greater response to SSRIs in women may be due to sex-specific biological differences (Gougoulaki et al., 2021). A study comparing escitalopram with citalopram in patients with dementia-related agitation found that both medications significantly reduced agitation (Pollock et al., 2007). A small, randomized study investigating escitalopram and citalopram also found that both interventions reduced agitation in different clinical conditions (Barak et al., 2011). This finding supports our current result of treatment of female VaD and MVAD with citalopram and escitalopram.

Moreover, dementia often exhibits more than one underlying pathology, known as MVAD, and there is no cure for the underlying illness of VaD and MVAD (DeTure and Dickson, 2019). SSRIs medications such as citalopram and escitalopram can attenuate symptoms by inhibiting neuronal nicotinic acetylcholine receptors (nAChR) subtypes by ion channel blockade and induction of receptor desensitization (Arias et al., 2021). Therefore, SSRI-induced inhibition of different nAChRs expressed the different neurotransmitter systems of the brain broadens the complexity by which citalopram and escitalopram may act clinically with an improved tolerability and safety profile for female VaD and MVAD patients (Yevtushenko et al., 2007).

We observed that memantine was associated with male AD, VaD, and MVAD patients. Memantine is a low-affinity non-competitive NMDA receptor antagonist and an approved medication for the treatment of cognitive symptoms of AD (Kikuchi, 2020). Therefore, our finding supports existing studies (Scarpini et al., 2003; Parsons et al., 2013) that memantine can be used for the treatment of cognitive functions in dementia patients. Memantine is reported to block the effect of *N*-methyl-D-aspartate (NMDA) receptor on neurons, and inhibit microglia activation to reduce pro-inflammatory factor production, such as extracellular superoxide anion, intracellular reactive oxygen species, nitric oxide, prostaglandin E_2_, and tumor necrosis factor-α (Johnson and Kotermanski, 2006; Wu et al., 2009; Parsons et al., 2013). The neuroprotective effect of memantine is thought to reduce β-amyloid peptide-induced neurodegeneration, as well as prevents the progression of neuronal loss (Wenk et al., 2006; Alley et al., 2010; Tanqueiro et al., 2018). Therefore, memantine may directly or indirectly affect the clearance or accumulation of Aβ species in the brain. Although the mechanism involves is not fully understood, it is possible that memantine prevents or breaks the aggregation of Aβ monomer into oligomeric forms and, subsequently, into Aβ plaques. This possibility is supported by the observation that memantine treatment in severe mice model of AD led to significantly increased soluble Aβ_1 − 42_ levels but significantly reduced the levels of insoluble Aβ and hippocampal amyloid deposition (Martinez-Coria et al., 2010). Interestingly, the levels of Aβ oligomer, was reduced in memantine-treated mice in the severe pathology group of AD animal models (Martinez-Coria et al., 2010; Figueiredo et al., 2013). Moreover, the levels of fibrillar oligomers were reduced in memantine-treated females, and there was no difference in cognitive performance between the two sexes (Martinez-Coria et al., 2010; Figueiredo et al., 2013). This effect was attributed to the relatively higher plasma memantine levels in females than in males (Figueiredo et al., 2013). While our current data cannot explain why females with AD, VaD, and MVAD were not treated with memantine, memantine can be used alone or in addition to ChEIs in patients with AD (Scarpini et al., 2003). While ChEIs block enzymes that metabolize acetylcholine and increase its levels (Ferreira-Vieira et al., 2016), memantine may prevent glutamate-induced neuronal damage by non-competitive antagonism of the NMDA receptor (Kutzing et al., 2012). This specific combination delays the progression of dementia by suppressing the activation of NMDA receptors (Parsons et al., 2013); however, they do present with side effects such as nausea, dizziness, asthenia, and anorexia that are associated with cholinergic overstimulation (Imbimbo, 2001). Therefore, the adverse events of memantine alone or with ChEIs may outweigh the benefits (Bailey-Taylor et al., 2022) in female AD, VaD, and MVAD patients.

Our results also showed that African American males with a history of ETOH and tobacco use and an increased length of stay were associated with AD, VaD, and MVAD. In contrast, Hispanic females were associated with VaD. This finding is supported by other studies that minority males and females with ETOH use, increasing length of stay for treatment, and increasing age present with higher rates of dementia (Langa et al., 2017). The higher rates of dementia among minorities are reported to be associated with an increased length of stay for care (Hill et al., 2015). While health disparities may be reduced by increasing and disease management among minorities (Chen and Zissimopoulos, 2018), there are other factors including socioeconomic and cultural factors. Health disparities often are mostly viewed through the lens of access to health care. However, the lack of diversity in clinical therapeutic development implies that to reduce disparities, it is also important to improve therapeutic targets for the large proportions in the minority population who are more affected by AD and VaD and MVAD. Prospective studies on factors that are associated with AD and VaD and MVAD among minorities should also focus on different treatment options for minorities by recruiting a diverse pool of participants into clinical trials of existing or new therapeutics.

## 5. Conclusion

Pharmacologic targets for males and females with AD, VaD, and MVAD show similarities and differences regarding treatment with specific AChEIs, SSRIs, and SGAs. Since males and females with AD, VaD, and MVAD present with differences in cognitive progression, effective therapeutic management of dementia patients should consider improved strategies in pharmacologic treatments to eliminate identified sex differences in the treatment of dementia for males and females.

## 6. Limitations

This retrospective data analysis was from a single institution, so the data cannot be generalized to other institutions and populations. Additionally, electronic medical records (EMR) were used for the data analysis, limiting the patient information that we could analyze. For example, there was no access to Mini-Mental State Examination (MMSE) data, medication therapy length, dosages, behavioral and psychological diagnoses, or a condition history to explain the length of hospital stay, whether it be dementia-related or related to another comorbidity. Along with these limitations, electronic medical records (EMRs) allow for the possibility of human error to limit the efficacy of the results. In addition to these limitations, this study uses terms to describe sex interchangeably, as the EMR data does not distinguish between them. Therefore, our findings may be due to biological differences between sexes, the social and cultural influence that gender has on an individual, or, more likely, a combination of the two. Further investigation into the distinction of results between sex and gender may further elucidate the cause of the findings and better inform changes for best clinical practices.

## Data availability statement

The raw data supporting the conclusions of this article will be made available by the authors, without undue reservation.

## Ethics statement

This is a retrospective data collection. This study was approved by the Institutional Review Board of PRISMA Health institutional committee for ethics (approval number: 00052571). All data were fully anonymized before they were accessed. Patients' data used in our retrospective analysis were from PRISMA Health Alzheimer's data registry. This study did not require informed consent for participation in accordance with the national legislation and institutional requirements.

## Author contributions

MS, NP, AM, SI-N, AI-N, RG, and TN designed the concept, experiment, and data collection. NP did the data analysis. RG, TN, and AI-N critically revised the drafts, interpreted the results, and read and approved the final version of the manuscript. All authors have read and approved the manuscript.
